# Perceptions and Preferences Regarding Opioid Sensor Devices: A Theory-Driven Cross-Sectional Survey of Community Responders and Healthcare Providers

**DOI:** 10.3390/healthcare14040498

**Published:** 2026-02-14

**Authors:** Bryson Grimsley, Shannon Woods, Madison Holland, Olivia Radzinski, Anne Taylor, Nicholas P. McCormick, Renee Delaney, Xinyu Zhang, Karen Marlowe, Lindsey Hohmann

**Affiliations:** 1 Department of Pharmacy Practice, Harrison College of Pharmacy, Auburn University, 1330 Walker Building, Auburn, AL 36849, USA; bug0001@auburn.edu (B.G.); sjw0096@auburn.edu (S.W.); mjh0088@auburn.edu (M.H.); ocr0006@auburn.edu (O.R.); marlokf@auburn.edu (K.M.); 2 Center for Opioid Research, Education, and Outreach (COACH), Harrison College of Pharmacy, Auburn University, 2316 Walker Building, Auburn, AL 36849, USA; ant0048@auburn.edu (A.T.); roberr7@auburn.edu (R.D.); 3 Department of Health Outcomes Research and Policy, Harrison College of Pharmacy, Auburn University, 4306 Walker Building, Auburn, AL 36849, USA; nzm0066@auburn.edu; 4 Department of Chemical Engineering, Samuel Ginn College of Engineering, Auburn University, 222 Foy Union Cir, Auburn, AL 36849, USA; xzz0004@auburn.edu

**Keywords:** opioids, opioid sensor device, Unified Theory of Acceptance and Use of Technology (UTAUT), healthcare provider, community responder

## Abstract

**Background/Objectives:** Identification of tools to minimize opioid-related harms is critical in the U.S. The purpose of this study was to better understand community responder and healthcare provider perceptions and preferences regarding the design and function of a potential new opioid sensor device (OSD). **Methods:** Adults aged ≥ 18 years employed as community responders or healthcare providers in Alabama were recruited via email to participate in an anonymous online cross-sectional survey informed by the Unified Theory of Acceptance and Use of Technology (UTAUT). Primary outcomes were assessed via multiple-choice and 7-point Likert-type scales (1 = strongly disagree, 7 = strongly agree) and included the following topics: (1) past OSD utilization (4 items); (2) perceived importance of OSD design elements (15 items); (3) OSD function and cost preferences (3 items); and (4) UTAUT measures including perceived usefulness of OSDs (3 items), ease of use (4 items), social factors (4 items), resources (4 items), concerns (3 items), and intentions (3 items). Differences in UTAUT measures across professions were assessed via Mann–Whitney U tests, and predictors of OSD utilization intention were analyzed via multiple linear regression. **Results:** Respondents (*N* = 145) included pharmacists (40.0%), nurses (23.4%), physicians (14.5%), behavioral health (4.8%), social work (4.8%), and law enforcement (0.7%). Availability in hospital emergency departments was rated as the most important device element (mean [SD] score: 6.66 [0.80]), followed by sensitivity and specificity of the test (6.42 [0.98]), rapid detection time (6.42 [0.88]), ability to detect opioids in a broad range of substance (6.42 [0.93]), and availability in law enforcement offices (6.33 [1.08]). A 2–5 min detection time was rated as reasonable by 32.6% of respondents, with 53.0% preferring to pay <USD 15 per test. There were no statistically significant differences in UTAUT scale scores across professions. Perceived usefulness (β = 0.493; *p* < 0.001), social acceptance (β = 0.281; *p* = 0.023), and resource availability (β = 0.708; *p* = 0.002) were positive predictors and perceived ease of use was a negative predictor (β = −0.472; *p* = 0.007) of intention to use an OSD. **Conclusions:** Newly developed OSDs should consider prioritizing accessibility in hospital emergency departments and law enforcement offices, ability to detect a broad range of opioids, detection time between 2 and 5 min, and cost less than USD 15 per test. Future research may explore perspectives from a more diverse sample across multiple states and different professional roles.

## 1. Introduction

The opioid crisis remains one of the most pressing public health challenges in the United States. In 2023 alone, the Centers for Disease Control and Prevention (CDC) reported around 80,000 opioid-related overdose deaths, underscoring the urgent need for innovative mitigation strategies [1]. Opioid misuse spans diverse populations and professions, with first responders, healthcare providers, and social workers often on the frontlines of managing opioid-related harms [2,3,4]. Despite ongoing efforts, including public education, naloxone distribution, and prescription monitoring programs, opioid overdoses persist at alarming rates [5,6,7,8]. These challenges highlight the need for novel tools and technologies that can complement existing harm reduction strategies.

The U.S. Food and Drug Administration (FDA) has identified opioid sensor devices (OSDs) as a potentially transformative tool in detecting and preventing opioid-related harm [9]. Such devices, designed to rapidly detect the presence of opioids in substances or the environment, could offer significant benefits in both clinical and field settings [10,11,12]. For example, opioid sensors could aid healthcare providers in identifying contaminated drugs in emergency departments or enable law enforcement officers to avoid accidental exposure to potent synthetic opioids like fentanyl. However, despite the theoretical promise of these devices, there are currently few commercially available options. Current marketed OSD devices include patient-facing tools like fentanyl test strips (FTSs) to detect the presence of fentanyl in other substances [13] and non-patient-facing handheld devices like TruNarc^®^ (Thermo Fisher Scientific, Waltham, MA, USA) for law enforcement to analyze substances for the presence of multiple narcotics in the field [14]. Recent studies have also investigated potential new patient-facing wearable OSDs including skin patches, wrist-mounted sensors like the ODRevive^®^ wristband (ODRevive, Birmingham, AL, USA) [15], and sonar-based smartphone sensors and apps to detect and alert first responders to opioid overdose [16]. The DOVE^®^ (Altrumed LLC, Philadelphia, PA, USA), a device worn on the shoulder, is a new product under study to both detect and reverse (via naloxone injection) opioid overdose [17].

Furthermore, little is known about the specific design and functional preferences of intended end-users of OSDs. Understanding the perceptions and preferences of key stakeholders—including healthcare providers, law enforcement personnel, and social workers—is essential for the successful development and adoption of opioid sensor devices. User-centered design principles emphasize the importance of tailoring technological innovations to meet the needs and expectations of their users [18]. Without input from these stakeholders, there is a risk of developing devices that are impractical, inaccessible, or ineffective in real-world scenarios. However, previous studies have mostly focused on wearable opioid sensors for patients, assessing patient acceptability and design preferences; few studies have assessed healthcare provider and community responder perspectives [16].

This pilot study aims to address this gap by exploring the perceptions and preferences of interdisciplinary community responders and healthcare professionals regarding the design and functionality of opioid sensor devices [19]. Specifically, it seeks to identify critical factors influencing the adoption of these devices into routine practice in both clinical and community contexts. The findings from this study will offer valuable guidance for developers and policymakers seeking to design effective and user-friendly opioid sensor technologies, advancing innovative solutions that empower frontline responders and healthcare providers to reduce opioid-related harms.

## 2. Materials and Methods

### 2.1. Study Design and Recruitment

An anonymous online cross-sectional survey was conducted between April and May 2024. Adults aged 18 and older who were employed as community responders (law enforcement, social work, emergency medical technicians, and behavioral health specialists) or healthcare providers (physicians, physician assistants, nurses, nurse practitioners, dentists, dental hygienists, pharmacists, and pharmacy technicians) in the state of Alabama were eligible to participate. Individuals were invited via email through the Auburn University Harrison College of Pharmacy Center for Opioid Research, Education, and Outreach listserv, with three contact attempts (an original invitation email plus two reminders at 2-week intervals). Participants were entered into a lottery to win one of three USD 100 electronic gift cards.

### 2.2. Sample Size Calculation

A power calculation was conducted using G*Power software version 3.1.9.7 (Heinrich-Heine-Universität Düsseldorf, Düsseldorf, Germany) [20,21]. Using a Type I error probability of α = 0.05 and a medium effect size of f^2^ = 0.15, a minimum sample size of *n* = 103 was determined to be sufficient to assess the study’s primary outcome (predictors of OSD utilization intention) via multiple linear regression with 80% power.

### 2.3. Data Collection and Measures

The link to the online survey was distributed via email, and individuals consented to participate by clicking “NEXT” at the bottom of the first page of the survey subsequent to reading the study Information Letter. Survey responses were collected using the Qualtrics^®^ (Provo, UT, USA) online platform. The survey instrument was developed by the research team, with measures guided by the Unified Theory of Acceptance and Use of Technology (UTAUT) [22,23]. The UTAUT postulates that an individual’s perceptions of a new technology, including performance expectancy (usefulness), effort expectancy (ease of use), social influence (social support or culture), and facilitating conditions (resources) influence their behavioral intention (desire to use) and actual use of the technology [22]. UTAUT items were adapted for use in the current survey by altering question wording to apply to the design of a new opioid sensor device. The survey instrument was pre-tested for face and content validity by members of the authors’ departments (*n* = 4) and modified based on feedback prior to distribution.

Survey measures included the following aspects: (1) prior use of opioid detection devices (e.g., fentanyl test strips) (4 items); (2) perceived significance of opioid sensor design features (15 items); (3) preferences related to the function and cost of opioid sensors (3-items); and (4) UTAUT metrics, including perceived usefulness of rapid-alert opioid sensors (3 items), ease of use (4 items), social influences on use (4 items), workplace resources affecting usage (4 items), concerns regarding use (3 items), and intentions to use an opioid sensor (3 items). Importance of design features and UTAUT dimensions were assessed on a 7-point Likert scale (from 1 = strongly disagree to 7 = strongly agree), while past usage and function preferences were recorded using multiple-choice questions. The full survey instrument can be found in the Supplementary Materials File S1.

### 2.4. Data Analysis

Analyses were conducted to characterize outcome measures using descriptive statistics (frequencies, percentages, means, standard deviations). Likert-type scale items were summed and averaged to create overall mean item and scale scores where applicable. Items were reverse-coded as necessary prior to calculation of mean scale scores, such that higher scale averages indicated more of a concept (e.g., greater perceived usefulness, more concerns). Internal consistency of UTAUT scales was assessed using the Cronbach’s alpha statistic. Mean UTAUT scale scores were compared across professions including healthcare (categorized as physicians, physician assistants, nurses, nurse practitioners, dentists, dental hygienists, pharmacists, and pharmacy technicians) and community responders (categorized as law enforcement, emergency medical technicians, social work, and behavioral health specialists) via Mann–Whitney U tests (α = 0.05).

Additionally, multiple linear regression was conducted to assess predictors of OSD utilization intention (dependent variable). Two regression models were performed: (1) including only mean UTAUT scale scores as predictors (Model 1); and (2) including UTAUT domains as predictors and controlling for covariates of profession (healthcare or community responder), history of opioid use (Yes/No), experience with opioid use disorder (Yes/No), previous OSD utilization (Yes/No), sex, race/ethnicity, and age. All analyses were conducted using SPSS Statistics Software version 29 (IBM Corp, Armonk, NY, USA).

### 2.5. Ethics Approval

All study procedures were approved via exempt review by the corresponding authors’ Institutional Review Board (Protocol # 24-773 EX 2403), and all participants provided informed consent.

## 3. Results

### 3.1. Participant Characteristics

Of 2390 invited participants, 174 individuals accessed the survey (7.3% response rate), with 145 survey responses being completed (83.3% completion rate) (Table 1). The majority of respondents worked in healthcare related fields, most of whom were pharmacists or pharmacy technicians (40.0%), nurses or nurse practitioners (23.4%), and physicians or physician assistants (14.5%). A minority of respondents worked in behavioral health (4.8%), social work (4.8%), and law enforcement (0.7%), and none as first responders. The majority of respondents were female (73.1) with a mean age of 52.54 years.

### 3.2. Current and Past Utilization

Only 13.8% of respondents were aware that rapid-alert opioid sensor devices exist on the market, with very few reporting previous awareness of fentanyl test strips (10.2%) and TruNarc^®^ (3.4%) (Table 2). Only 2.1% of participants had ever utilized a rapid-alert opioid sensor device.

### 3.3. Perceived Importance and Preferences for Design Features

In terms of respondents’ perceptions of the importance of rapid-alert opioid sensor device elements (Table 3), availability in hospital emergency departments was the highest rated item with mean (SD) score of 6.66 (0.80) out of 7. The sensitivity and specificity of the test (mean [SD] score: 6.42 [0.98]), rapid detection time (6.42 [0.88]), ability to detect opioids in a broad range of substance (6.42 [0.93]), availability in law enforcement offices (6.33 [1.08]), ability to detect multiple types of opioids (6.29 [1.12]), and portability (6.20 [0.96]) were also highly rated. Availability for purchase in pharmacies with a physician’s prescription was the lowest-rated item (4.76 [1.71]).

Preferences regarding additional device features, including function, form, and price, were also assessed (Table 4). Specifically, participants most often reported that the greatest need for a rapid-alert opioid sensor device in their job would be for point-of-care testing or in-field diagnosis of opioid intoxication (48.3%), followed by checking unknown solid substances for presence of opioids (40.8%). Ensuring patient adherence to opioid therapy was the least frequently chosen use for the device (27.9%). Furthermore, 51.7% of respondents believed that a no-contact probe sensor would be the most useful form of the device for their job, while 42.9% believed test strips and 24.5% believed skin patches would be most useful. A detection time of between 2 and 5 min was rated as reasonable by the greatest proportion of respondents (32.6%), with 34.1% stating the maximum they would pay for a rapid-alert opioid sensor device kit was less than USD 100, and 53.0% preferring to pay less than USD 15 per test.

### 3.4. Factors Affecting Utilization

In terms of the UTAUT scales, internal consistency ranged from Cronbach’s alpha of 0.562 to 0.943 (Table 5). Overall, respondents were fairly positive regarding their readiness and ability to implement a new rapid-alert opioid sensor device into their workflow. In particular, perceived ease of use of a rapid-alert opioid sensor device was high with a mean (SD) overall scale score of 5.76 (1.00). Perceived availability of resources in the workplace to facilitate implementation of a rapid-alert opioid sensor device (mean [SD] overall scale score: 5.20 [0.98]), perceived supportive social influences in the workplace (4.96 [1.26]), and perceived usefulness (4.93 [1.45]) were moderately high and concerns (2.63 [1.32]) were low. Intentions to utilize a rapid-alert opioid sensor device in the next three months were neutral, with a mean (SD) overall scale score of 4.00 (1.61). Furthermore, mean (SD) OSD utilization intention tended to be lower among healthcare professionals (3.89 [1.62]; *n* = 120) compared to community responders (4.80 [1.14]; *n* = 15); however, this difference did not reach statistical significance (*p* = 0.060). There were no statistically significant differences in other UTAUT scale scores across professions.

### 3.5. Predictors of OSD Utilization Intention

After controlling for covariates, perceived usefulness (β = 0.493; *p* < 0.001), social acceptance (β = 0.281; *p* = 0.023), and resource availability (β = 0.708; *p* = 0.002) were positive predictors and perceived ease of use was a negative predictor (β = −0.472; *p* = 0.007) of intention to use an OSD (Table 6; Model 2). Furthermore, profession was a positive predictor of OSD utilization intention, such that community responders had higher intention compared to healthcare professionals. Statistical significance of findings was consistent across Model 1 and Model 2 in terms of UTAUT domains.

## 4. Discussion

This study aimed to fill a gap in the OSD literature by exploring the perceptions and preferences of interdisciplinary professionals regarding the design and functionality of these devices, with an eye toward developing a new user-friendly device. Of note, only a small percentage of respondents (2.1%) reported that they had ever used a rapid-alert opioid sensor device. Further, less than 14% were aware of rapid-alert opioid sensor devices available on the market, with awareness of patient-facing products like fentanyl test strips (FTSs) slightly higher than other non-patient-facing devices like TruNarc^®^ [14]. Although awareness of OSDs was low across the board, likely due to the emerging nature of many of these devices, this disparity in awareness based on device type may be due to media coverage surrounding the decriminalization of FTS in some U.S. states [24] as well as emerging educational programming for healthcare providers and law enforcement related to FTS [6]. However, there is a comparative dearth of media coverage and educational programming regarding non-patient-facing devices like TruNarc^®^, leading to gaps in community responder and healthcare professionals’ awareness and knowledge. This points to the need for targeted educational campaigns highlighting a variety of OSD device types (e.g., wearable sensors, in-field diagnostic devices, patient-facing harm reduction tools) [16] for professional end-users.

In terms of respondents’ perceived importance of rapid-alert opioid sensor device elements, access to the device in certain settings was a polarizing topic. Specifically, availability of OSDs in hospital emergency departments was the highest rated item. This is no surprise as many community responders and healthcare professionals are aware that opioid overdoses are a medical emergency and likely treated in the emergency department. Indeed, the Centers for Disease Control and Prevention (CDC) reports that nonfatal opioid-involved overdoses treated in the emergency departments are increasing and approximately 179/10,000 emergency medical services (EMSs) encounters involve nonfatal opioid overdoses [25]. Similarly, availability of OSDs in law enforcement offices was also perceived as highly important, aligning with previous research that indicates law enforcement officers have routinized opioid harm reduction services such as naloxone administration and treatment referrals into their core job roles [26]. In contrast, the perceived importance of availability of OSDs for purchase in pharmacies over-the-counter (OTC) was less highly rated, and purchase in pharmacies with a physician’s prescription was the lowest-rated item. This indicates a potential unawareness among community responders and healthcare professionals regarding the role of pharmacists at increasing access to opioid harm reduction tools, especially in rural or healthcare-disadvantaged areas experiencing hospital closures and a lack of traditional medical providers [27,28]. In these instances, pharmacies may represent the only readily available healthcare settings, presenting a unique opportunity for access to patient-facing OSDs [27]. This points to the need for future training and education of community responders and healthcare providers regarding interprofessional roles surrounding OSD utilization and access.

Additionally, other OSD features that were perceived as highly important in the current study revolved around functionality, specifically the sensitivity and specificity of the test, rapid detection time (2–5 min), ability to detect opioids in a broad range of substance, ability to detect multiple types of opioids, portability, and cost (less than USD 15 per test). Community responders and healthcare professionals also preferred an OSD in the form of a no-contact probe sensor or test strips, with few preferring wearable sensor devices like skin patches. This contrasts with the focus on wearable device format and design features investigated in the preponderance of OSD literature to date [16]. These perceptions may be leveraged to ensure relevance and feasibility of future newly developed OSDs marketed for use in the community responder and healthcare professional markets.

Factors that may affect participants’ use of an OSD were also assessed using the UTAUT framework. Interestingly, perceived ease of use and workplace resource availability were the most highly rated. However, perceived social acceptance and usefulness, while positive, were less highly rated. This finding may be driven by disparities at the item level within the social acceptance and usefulness constructs. Specifically, within the social acceptance construct, respondents believed that their workplace would support the use of an OSD and their professional colleagues would be helpful, but were less positive as to whether people who were important to them or influenced their behavior would support their use of an OSD. In terms of usefulness, participants believed that they would find a rapid-alert opioid sensor useful in their job, but were less positive as to whether an OSD would enable them to increase their productivity or accomplish their tasks more quickly. Future studies may further investigate these nuanced differences in perceived social acceptance and usefulness of OSDs. Additionally, given that the majority of participants had never used an OSD, perceptions of ease of use and usefulness were based on hypothetical descriptions rather than direct experience, which may not reflect actual behavior upon implementation. Thus, follow-up studies at future points in time after OSDs become more available on the commercial market and implemented in routine practice may be needed.

Further, although concerns were low, intention to utilize an OSD in the next three months was neutral overall. This is dissimilar to previous research in Alabama that found pharmacists’ intentions to provide fentanyl test strips were positive [29]. Future research may wish to investigate differences in OSD utilization, provision, and recommendation intentions for a variety of OSD formats and designs (e.g., patient-facing, wearable devices, no-contact probes) and across a variety of healthcare professionals. Of note, opioid sensor device utilization intention tended to be slightly higher amongst community responders versus healthcare professionals in bivariate analyses, although this finding was not statistically significant and must be interpreted with caution. However, regression analyses revealed profession to be a statistically significant positive predictor of OSD utilization intention, with community responders more likely to have higher intention compared to healthcare professionals. Future studies may wish to investigate this potential difference amongst a sample with a larger number of community responders, given that the split between groups was unbalanced in the current study with the majority of respondents being healthcare professionals. Additionally, although perceived usefulness, social acceptance, and resource availability were positive predictors of OSD utilization intention, it is unexpected that perceived ease of use was a negative predictor. It may be that those who perceive an OSD device as “too easy” may assign decreased value to the device due to a perception of lower quality [30], leading to less of a desire to obtain or utilize the device. However, a conceptual misunderstanding among participants regarding the “ease of use” construct cannot be ruled out, and may be clarified with more extensive pre- and pilot-testing of the survey instrument in future studies. Further, variables or covariates that were not measured may have impacted the regression analyses; future research may consider measuring additional constructs shown to affect valuation, such as overconfidence [31], and assessing the association of task complexity with perceived device value [31].

### Limitations

This pilot study was limited to healthcare providers, law enforcement, social workers, and behavioral health specialists in Alabama. Findings may not be generalizable to other U.S. states; however, perceptions may be applicable to other Deep South states with similar opioid overdose rates. Furthermore, challenges were encountered with recruitment of community responders including first responders, law enforcement officers, social workers, and behavioral health specialists, leading to an unbalanced sample more heavily weighted towards healthcare professionals. This uneven distribution limits the generalizability of the findings regarding preferences for opioid sensor devices across all intended user groups, and limits the power of the Mann–Whitney U comparisons across professions. Thus, comparisons across professional groups should be considered exploratory and interpreted with caution, and future studies should assess perceptions amongst a sample with a larger proportion of community responders. Recruitment across multiple states, utilizing multimodal recruitment strategies such as social media and direct mailing, and more clearly defining the “Other” category when characterizing profession would enhance recruitment among this group and strengthen the relevance of findings among diverse professional contexts. In addition, the survey sample was characterized by a relatively high mean age (52.54 years), decreasing the generalizability of the findings and potentially influencing perceptions and preferences surrounding OSD adoption and design [32]. Future studies may wish to purposively recruit a younger sample and assess differences in perceptions and preferences across age groups.

Furthermore, the overall survey response rate was low (7.3%), and data was collected via participant self-report, introducing the potential for non-response bias and social desirability bias. While the latter was mitigated by the anonymous nature of the survey, future studies should employ multimodal recruitment strategies (as described above) to enhance overall recruitment and improve survey response rates. Further, the study was cross-sectional in nature and looked at preferences and perceptions at a single point in time. Perceptions regarding opioid sensor technology may change as these devices become more known and used by professionals. Future studies may consider assessing OSD preferences and perceptions across multiple points in time. Additionally, the internal consistency of the resource scale (Cronbach’s alpha = 0.562) was comparatively lower than the other scales and below the typical threshold of 0.7 [33]. Removal of the reverse-coded item *“A rapid-alert opioid sensor would not be compatible with other systems I use”* would increase the Cronbach’s alpha to 0.723. However, based on recommendations by Hussey [34] and Raykov [35], the authors chose to retain this item in the scale due to its conceptual value, thus maintaining content validity and increasing comparability to other published studies that adapted items from the validated UTAUT measures published by Venkatesh et al. [22]. To further ensure the content validity of the survey instrument, future studies may wish to expand upon this pilot work by undertaking more extensive survey pre-testing (which was limited to four individuals in the current study) and pilot-testing.

## 5. Conclusions

This study provides insight into the awareness and preferences of Alabama community responders and healthcare professionals regarding the use of rapid-alert opioid sensor devices. Although awareness of opioid sensor devices was low, perceived ease of use and usefulness were positively rated. Based on end-user preferences, newly developed opioid sensor devices should consider prioritizing accessibility in hospital emergency departments and law enforcement offices, ability to detect a broad range of opioids, detection time between 2 and 5 min, and cost less than USD 15 per test. Future research may explore perspectives from a more diverse sample across multiple states and different professional roles.

## Figures and Tables

**Table 1:** Participant characteristics (*n* = 145).

Characteristics	*n* (%) ^a^
Profession	
Dentist	7 (4.8)
Dental hygienist	-
Nurse	17 (11.7)
Nurse practitioner	17 (11.7)
Pharmacist	52 (35.9)
Pharmacy technician	6 (4.1)
Physician	20 (13.8)
Physician assistant	1 (0.7)
Emergency medical technician (EMT)/first responder	-
Law enforcement	1 (0.7)
Social worker	7 (4.8)
Behavioral health specialist	7 (4.8)
Other	10 (6.9)
Are you receiving or are you a friend/family member of someone receiving opioid therapy (e.g., morphine, hydrocodone) for chronic pain?	
Yes	20 (13.8)
No	125 (86.2)
Have you or someone you know been personally or professionally affected by opioid use disorder or opioid misuse?	
Yes	97 (66.9)
No	48 (33.1)
What is your sex?	
Male	37 (25.5)
Female	106 (73.1)
With which race or ethnicity do you most closely identify?	
American Indian or Alaska Native	1 (0.7)
Asian	-
Black or African American	18 (12.4)
Hispanic or Latino(a)	5 (3.4)
White	114 (78.6)
More than one race/ethnicity	2 (1.4)
	**Mean (SD)**
Age, years	52.54 (13.727)

^a^ Frequencies and percentages may differ due to item non-response.

**Table 2:** Current and past utilization of opioid sensors (*n* = 145).

Questions	*n* (%) ^a^
Are you aware of any devices that can rapidly sense the presence of opioids in a substance in real-time (a rapid-alert opioid sensor device)?	
Yes	20 (13.8)
No	125 (86.2)
Which rapid-alert opioid sensor devices have you previously heard about? Please select all that apply.	
TruNarc	5 (3.4)
Fentanyl test strips	15 (10.2)
ODRevive	1 (0.7)
Have you ever utilized a rapid-alert opioid sensor device?	
Yes	3 (2.1)
No	141 (97.9)
In the past 3 months, how frequently or infrequently did you utilize a rapid-alert opioid sensor device?	
Never	142 (98.6)
Rarely	1 (0.7)
Sometimes	-
Often	1 (0.7)
All the time	-

^a^ Frequencies and percentages may differ due to item non-response.

**Table 3:** Perceived importance regarding features of a rapid-alert opioid sensor device (*n* = 145) ^a^.

Features	*n* (%)							Mean (SD)
	1	2	3	4	5	6	7	
Affordability	1 (0.8)	2 (1.5)	4 (3.0)	9 (6.8)	19 (14.4)	38 (28.8)	59 (44.7)	5.98 (1.257)
Portability	1 (0.8)	-	1 (0.8)	6 (4.5)	11 (8.3)	55 (41.7)	58 (43.9)	6.20 (0.963)
Sensitivity and specificity of the test	1 (0.8)	-	1 (0.8)	6 (4.6)	7 (5.3)	34 (26.0)	82 (62.6)	6.42 (0.976)
Ability to detect opioids in a broad range of substances	1 (0.8)	-	1 (0.8)	4 (3.0)	7 (5.3)	41 (31.1)	78 (59.1)	6.42 (0.925)
Ability to detect multiple types of opioids	1 (0.8)	1 (0.8)	3 (2.3)	6 (4.5)	7 (5.3)	39 (29.5)	75 (56.8)	6.29 (1.116)
Ability to detect the amount of an opioid in a substance (potency and purity)	1 (0.8)	2 (1.5)	5 (3.8)	18 (13.6)	26 (19.7)	37 (28.0)	43 (32.6)	5.64 (1.314)
Rapid detection time	-	-	2 (1.5)	5 (3.8)	7 (5.3)	39 (29.8)	78 (59.5)	6.42 (0.877)
No contact with unidentified drug substances (a “no-contact” probe)	-	3 (2.3)	1 (0.8)	33 (25.0)	14 (10.6)	28 (21.2)	53 (40.2)	5.68 (1.361)
Ability to remotely view results using a mobile app	-	5 (3.8)	5 (3.8)	25 (19.1)	25 (19.1)	32 (24.4)	39 (29.8)	5.46 (1.388)
Ability to remotely view results using a computer-based web interface	-	8 (6.1)	5 (3.8)	30 (22.7)	25 (18.9)	33 (25.0)	31 (23.5)	5.23 (1.440)
Coverage by patient health insurance	-	4 (3.0)	6 (4.5)	21 (15.9)	24 (18.2)	33 (25.0)	44 (33.3)	5.58 (1.371)
Availability for purchase in pharmacies as an over-the-counter (OTC) product	3 (2.3)	2 (1.5)	5 (3.8)	17 (12.9)	16 (12.1)	42 (31.8)	47 (35.6)	5.69 (1.436)
Availability for purchase in pharmacies with a physician’s prescription	6 (4.5)	9 (6.8)	14 (10.6)	33 (25.0)	12 (9.1)	36 (27.3)	22 (16.7)	4.76 (1.708)
Availability in law enforcement offices	1 (0.8)	-	1 (0.8)	10 (7.6)	11 (8.3)	26 (19.7)	83 (62.9)	6.33 (1.082)
Availability in hospital emergency departments	-	1 (0.8)	1 (0.8)	2 (1.5)	5 (3.8)	20 (15.2)	103 (78.0)	6.66 (0.799)

^a^ Participants were queried to answer the prompt, *“To me, the most important parts of a rapid-alert opioid sensor are…”* on a scale of 1–7, where 1 = strongly disagree, 2 = disagree, 3 = somewhat disagree, 4 = neutral, 5 = somewhat agree, 6 = agree, and 7 = strongly agree.

**Table 4:** Preferences regarding features of a rapid-alert opioid sensor device (*n* = 145).

Questions	*n* (%) ^a^
For your job, in which circumstances would you use a rapid-alert opioid sensor device? Please select all that apply.	
Ensuring patient adherence to opioid therapy	41 (27.9)
Monitoring patients remotely for signs of opioid misuse (“real-time” monitoring)	53 (36.1)
Checking unknown solid substances (e.g., powders, pills) for the presence of opioids	60 (40.8)
Checking biological fluids (e.g., urine or blood) for the presence of opioids	46 (31.3)
Point-of-care testing or in-field diagnosis of opioid intoxication	71 (48.3)
What form of device would be most useful to you in your workplace? Please select all that apply.	
No-contact probe sensor	76 (51.7)
Test strips	63 (42.9)
Skin patches	36 (24.5)
What would be a reasonable detection time for a rapid-alert opioid sensor? Detection time is defined as the amount of time needed for the device to sense and display results regarding the presence, identity, and amount of opioids detected.	
1 min	30 (22.7)
2 min	43 (32.6)
5 min	43 (32.6)
15 min	13 (9.8)
30 min	3 (2.3)
45 min	-
60 min	-
What is the MAXIMUM you would pay to purchase a rapid-alert opioid sensor device kit to use in your workplace or home? The device kit may take the form of a probe sensor to identify unknown substances, test strips for point-of-care diagnosis, and/or skin patches for patient use. The device kit will include all technology needed to monitor and interpret results.	
<USD 100	45 (34.1)
USD 100	35 (26.5)
USD 200	19 (14.4)
USD 300	13 (9.8)
USD 400	4 (3.0)
USD 500	13 (9.8)
>USD 500	3 (2.3)
What is the MAXIMUM you would pay per test for a rapid-alert opioid sensor device? For example, if the device kit came with enough testing supplies (e.g., disposable test strips) for at least one month, what is the maximum you would pay per test?	
<USD 15 per test	70 (53.0)
USD 20 per test	46 (34.8)
USD 30 per test	10 (7.6)
USD 40 per test	3 (2.3)
>USD 40 per test	3 (2.3)

^a^ Frequencies and percentages may differ due to item non-response.

**Table 5:** Factors affecting utilization of a rapid-alert opioid sensor (*n* = 145) ^a^.

Questions	Mean (SD)
***Usefulness (Cronbach’s Alpha = 0.877)***	***4.93 (1.448)***
I would find a rapid-alert opioid sensor useful in my job.	5.58 (1.454)
Using a rapid-alert opioid sensor would enable me to accomplish tasks more quickly.	4.78 (1.706)
Using a rapid-alert opioid sensor would increase my productivity.	4.29 (1.698)
***Ease of Use (Cronbach’s Alpha = 0.868)***	***5.76 (0.996)***
My interaction with a rapid-alert opioid sensor would be clear and understandable.	5.56 (1.301)
It would be easy for me to become skillful at using a rapid-alert opioid sensor.	5.98 (1.095)
I would find a rapid-alert opioid sensor easy to use.	5.67 (1.177)
Learning to operate a rapid-alert opioid sensor would be easy for me.	5.83 (1.116)
***Social Factors (Cronbach’s Alpha = 0.795)***	***4.96 (1.263)***
People who influence my behavior would think that I should use a rapid-alert opioid sensor.	4.27 (1.752)
People who are important to me would think that I should use a rapid-alert opioid sensor.	4.25 (1.743)
My professional colleagues would be helpful in the use of a rapid-alert opioid sensor.	5.51 (1.338)
In general, my workplace would support the use of a rapid-alert opioid sensor.	5.51 (1.418)
***Resources (Cronbach’s Alpha = 0.562)***	***5.20 (0.979)***
I would have the resources necessary to use a rapid-alert opioid sensor.	5.31 (1.296)
I would have the knowledge necessary to use a rapid-alert opioid sensor.	5.86 (1.150)
A rapid-alert opioid sensor would not be compatible with other systems I use. ^b^	3.61 (1.413)
A specific person (or group) would be available for assistance with rapid-alert opioid sensor difficulties.	5.11 (1.443)
***Concerns (Cronbach’s Alpha = 0.866)***	***2.63 (1.322)***
I would feel apprehensive about using a rapid-alert opioid sensor.	2.71 (1.510)
I would hesitate to use a rapid-alert opioid sensor for fear of making mistakes I cannot correct.	2.60 (1.424)
A rapid-alert opioid sensor would be somewhat intimidating to me.	2.52 (1.453)
***Intentions (Cronbach’s Alpha = 0.943)***	***4.00 (1.611)***
I intend to use a rapid-alert opioid sensor in the next 3 months.	4.27 (1.611)
I predict I will use a rapid-alert opioid sensor in the next 3 months.	3.95 (1.772)
I plan to use a rapid-alert opioid sensor in the next 3 months.	3.72 (1.670)

^a^ Participants were queried to rate their level of agreement or disagreement regarding factors that affect/would affect their use of a rapid-alert opioid sensor device in their workplace on a scale of 1–7, where 1 = strongly disagree, 2 = disagree, 3 = somewhat disagree, 4 = neutral, 5 = somewhat agree, 6 = agree, and 7 = strongly agree. ^b^ Items were reverse-coded prior to calculating the mean scale score.

**Table 6:** Predictors of OSD utilization intention (*n* = 145) ^a^.

Predictors	Β	95% CI	*p*-Value
^b^ Model 1: R^2^ = 0.392, F(df) = 12.123(5), p < 0.001 *			
Usefulness	0.425	0.205, 0.646	<0.001 *
Ease of use	−0.399	−0.728, −0.069	0.018 *
Social influences	0.276	0.042, 0.510	0.021 *
Resource availability	0.699	0.298, 1.100	<0.001 *
Concerns	0.116	−0.098, 0.330	0.284
^c^ Model 2: R^2^ = 0.474, F(df) = 6.014(12), p < 0.001 *			
Usefulness	0.493	0.264, 0.723	<0.001 *
Ease of use	−0.472	−0.811, −0.133	0.007 *
Social influences	0.281	0.039, 0.522	0.023 *
Resource availability	0.708	0.263, 1.152	0.002 *
Concerns	0.158	−0.069, 0.386	0.169
History of opioid use (Ref = Yes)	0.008	−0.852, 0.869	0.985
Experience with opioid use disorder (Ref = Yes)	−0.463	−1.016, 0.089	0.099
Previous OSD utilization (Ref = Yes)	1.717	−0.360, 3.793	0.104
Sex (Ref = Male)	−0.152	−0.802, 0.499	0.644
Race/ethnicity (Ref = white)	−0.339	−1.021, 0.343	0.326
Age	−0.010	−0.032, 0.012	0.382
Profession (Ref = Healthcare)	1.013	0.092, 1.934	0.032 *

Statistical significance at the α = 0.05 level denoted by *. ^a^ Multiple linear regression to assess predictors of OSD utilization intention (dependent variable). ^b^ Model 1 (unadjusted model): including only mean UTAUT scale scores as predictors. ^c^ Model 2 (adjusted model): including UTAUT domains as predictors and controlling for covariates of profession (healthcare versus community responder), history of opioid use (Yes/No), experience with opioid use disorder (Yes/No), previous OSD utilization (Yes/No), sex (male versus female), race/ethnicity (Non-Hispanic white versus American Indian or Alaska Native, Asian, Black or African American, Hispanic or Latino(a), or Multiracial), and age. Model assumptions were met, including multivariate normality (as indicated by the histogram and normal P-P plot of standardized residuals), collective linearity (as indicated by the scatterplot of standardized residuals versus predicted values), no autocorrelation (Durbin–Watson = 1.867), no multicollinearity (VIF < 5), no influential outliers (Cook’s Distance < 1), and no heteroskedasticity (White Test for Heteroskedasticity *p* = 0.305).

## Data Availability

The datasets generated and/or analyzed during the current study are not publicly available due to restrictions within the Institutional Review Board protocol.

## Supplementary Materials

The following supporting information can be downloaded at: https://www.mdpi.com/article/10.3390/healthcare14040498/s1, File S1: Survey Instrument.

## Abbreviations

The following abbreviations are used in this manuscript:

CDC	Centers for Disease Control and Prevention
FDA	Food and Drug Administration
OSD	opioid sensor device
SD	standard deviation

## References

[1] Ahmad F.B., Cisewski J.A., Rossen L.M., Sutton P. (2025). Provisional Drug Overdose Data Death Counts. National Center for Health Statistics. https://www.cdc.gov/nchs/nvss/vsrr/drug-overdose-data.htm.

[2] Bolshakova M., Bluthenthal R., Sussman S. (2019). Opioid use and misuse: Health impact, prevalence, correlates and interventions. Psychol. Health.

[3] Chisholm-Burns M.A., Spivey C.A., Sherwin E., Wheeler J., Hohmeier K. (2019). The opioid crisis: Origins, trends, policies, and the roles of pharmacists. Am. J. Health-Syst. Pharm..

[4] Lofaro R.J., Sapat A. (2024). Occupational and Personal Challenges During the Opioid Crisis: Understanding First Responders’ Experiences and Viewpoints of Clients with Opioid Use Disorder. Disaster Med. Public Health Prep..

[5] Devries J., Rafie S., Polston G. (2017). Implementing an overdose education and naloxone distribution program in a health system. J. Am. Pharm. Assoc..

[6] Hohmann L., Phillippe H., Marlowe K., Jeminiwa R., Hohmann N., Westrick S., Fowler A., Fox B. (2022). A state-wide education program on opioid use disorder: Influential community members’ knowledge, beliefs, and opportunities for coalition development. BMC Public Health.

[7] Nielsen S., Horn F., McDonald R., Eide D., Walley A.Y., Binswanger I., Langford A.V., Prathivadi P., Wood P., Clausen T. (2024). Development of pharmacy-based best practices to support safer use and management of prescription opioids based on an e-Delphi methodology. Res. Soc. Adm. Pharm..

[8] Wilkerson D.M., Groves B.K., Mehta B.H. (2020). Implementation of a naloxone dispensing program in a grocery store–based community pharmacy. Am. J. Health-Syst. Pharm..

[9] United States Food and Drug Administration (2019). FDA Innovation Challenge: Devices to Prevent and Treat Opioid Use Disorder.

[10] Teck J.T.W., Oteo A., Baldacchino A. (2023). Rapid opioid overdose response system technologies. Curr. Opin. Psychiatry.

[11] Mesa J.C., MacLean M.D., María M., Nguyen A., Patel R., Diemer T., Lim J., Lee C.H., Lee H. (2023). A Wearable device towards automatic detection and treatment of opioid overdose. IEEE Trans. Biomed. Circuits Syst..

[12] Ciatti J.L., Vázquez-Guardado A., Brings V.E., Park J., Ruyle B., Ober R.A., McLuckie A.J., Talcott M.R., Carter E.A., Burrell A.R. (2024). An autonomous implantable device for the prevention of death from opioid overdose. Sci. Adv..

[13] DanceSafe New Fentanyl Test Strips. https://dancesafe.org/product/fentanyl-test-strips-pack-of-10-free-shipping/.

[14] Thermo Scientific TruNarc™ Handheld Narcotics Analyzer. https://www.thermofisher.com/order/catalog/product/TRUNARC.

[15] ODRevive Engineering Faster Overdose Responses. https://www.odrevive.com/.

[16] Oteo A., Daneshvar H., Baldacchino A., Matheson C. (2023). Overdose alert and response technologies: State-of-the-art review. J. Med. Internet Res..

[17] Lingamoorthy A., Watson A., Henderson K., Mandal A., Gordon D., Ma X., Weimer J., Kandasamy N., Brenner J.S. Dove: Shoulder-Based Opioid Overdose Detection and Reversal Device. Proceedings of the 2023 IEEE/ACM Conference on Connected Health: Applications, Systems and Engineering Technologies (CHASE).

[18] Walden A., Garvin L., Smerek M., Johnson C. (2020). User-centered design principles in the development of clinical research tools. Clin. Trials.

[19] Hohmann L., Marlowe K., Delaney R., Zhang X. (2025). A Cross-Sectional Survey Assessing Law Enforcement and Healthcare Provider Perceptions and Preferences Surrounding an Opioid Sensor Device [Abstract]. J. Am. Pharm. Assoc..

[20] Heinrich Heine Universitat G*Power Manual. https://www.psychologie.hhu.de/fileadmin/redaktion/Fakultaeten/Mathematisch-Naturwissenschaftliche_Fakultaet/Psychologie/AAP/gpower/GPowerManual.pdf.

[21] Kang H. (2021). Sample size determination and power analysis using the G*Power software. J. Educ. Eval. Health Prof..

[22] Venkatesh V., Morris M.G., Davis G.B., Davis F.D. (2003). User acceptance of information technology: Toward a unified view. MIS Q..

[23] Ammenwerth E., Scott P. (2019). Technology acceptance models in health informatics: TAM and UTAUT. Applied Interdisciplinary Theory in Health Informatics.

[24] Sell M. Fentanyl Testing Strips Now Legal in Alabama. https://aldailynews.com/fentanyl-testing-strips-now-legal-in-alabama/.

[25] Casillas S., Pickens C., Stokes E., Walters J., Vivolo-Kantor A. (2022). Patient-Level and County-Level Trends in Nonfatal Opioid-Involved Overdose Emergency Medical Services Encounters—491 Counties, United States, January 2018–March 2022. Morb. Mortal. Wkly. Rep. (MMWR).

[26] Pike E., Tillson M., Staton M., Webster J.M. (2021). The role of law enforcement officers in responding to the opioid epidemic: A qualitative assessment. Subst. Abus..

[27] Zahnd W. (2023). Rural Pharmacies Provide Multi-Faceted Value to Rural Communities. The Rural Monitor.

[28] Chatterjee P. (2022). Causes and consequences of rural hospital closures. J. Hosp. Med..

[29] Woods S., Blythe E., Valle-Ramos G., Richardson J., Pham K., Diggs K., Harris K., Zhao Y., Hohmann L. (2025). Alabama community pharmacists’ knowledge and perceptions regarding fentanyl test strips: A cross-sectional survey. J. Am. Pharm. Assoc..

[30] Jackson J. Lower Prices, Sell More? Debunking Pricing Myths. https://www.cim.co.uk/content-hub/blog/lower-prices-sell-more-debunking-the-pricing-myth/.

[31] Lee C.-C., Lee H.-Y., Yeh W.-C., Yu Z. (2022). The impacts of task complexity, overconfidence, confirmation bias, customer influence, and anchoring on variations in real estate valuations. Int. J. Strateg. Prop. Manag..

[32] Fahmiyah I., Utami I.Q., Ningrum R.A., Fakhruzzaman M.N., Pratama A.I., Triangga Y.M. (2023). Examining the effect of teacher’s age difference on learning technology adoption using technology acceptance model. Proceedings of the International Conference on Advanced Technology and Multidiscipline (ICATAM) 2021:“Advanced Technology and Multidisciplinary Prospective Towards Bright Future” Faculty of Advanced Technology and Multidiscipline.

[33] Tavakol M., Dennick R. (2011). Making sense of Cronbach’s alpha. Int. J. Med. Educ..

[34] Hussey I., Alsalti T., Bosco F., Elson M., Arslan R.C. (2023). An aberrant abundance of Cronbach’s alpha values at. 70. Adv. Methods Pract. Psychol. Sci..

[35] Raykov T. (2008). Alpha if item deleted: A note on loss of criterion validity in scale development if maximizing coefficient alpha. Br. J. Math. Stat. Psychol..

