# Supplementary material for: Perceptions and Preferences Regarding Opioid Sensor Devices: A Theory-Driven Cross-Sectional Survey of Community Responders and Healthcare Providers
Source: Healthcare (Basel). 2026 Feb 14;14(4):498. doi: 10.3390/healthcare14040498 (PMC12940477; doi:10.3390/healthcare14040498)
Supplement: Supplementary file 1 [file healthcare-14-00498-s001.zip › healthcare-4076999-supplementary.pdf]

SUPPLEMENTAL MATERIAL: SUPPLEMENTAL FILE S1 – SURVEY INSTRUMENT

Perceptions Regarding a Real-Time Opioid Sensor Device
Law Enforcement, First Responder, and Healthcare Provider Survey

Thank you for your participation in this project! The purpose of this research study is to better understand law enforcement, first responder, and healthcare provider perceptions and preferences regarding the design and function of a potential new rapid-alert opioid sensor device that is being developed. This rapid-alert opioid sensor device will be able to detect and identify opioids (e.g., morphine, hydrocodone, fentanyl) present in drug substances or the human body in real-time. The findings from this survey will inform the device development process. This survey consists of 4 sections and should take about 20 minutes to complete. All responses will remain anonymous.

The Information Letter with more details regarding this research study can be reviewed here: [link to Information Letter]

Clicking the "NEXT" button below serves as your consent to participate in this survey study.

I. INFORMATION ABOUT YOU

1. Please indicate your profession:
☐ Dentist ☐ Dental hygienist ☐ Nurse (RN, LPN) ☐ Nurse practitioner
☐ Pharmacist ☐ Pharmacy technician ☐ Physician ☐ Physician assistant (PA)
☐ EMT / first responder ☐ Law enforcement ☐ Social worker ☐ Behavioral health specialist
☐ Student. Please specify: ________________ ☐ Other. Please specify: __________________

2. Are you receiving or are you a friend / family member of someone receiving opioid therapy (e.g., morphine, hydrocodone) for chronic pain?
☐ Yes ☐ No ☐ Prefer not to answer

3. Have you or someone you know been personally or professionally affected by opioid use disorder or opioid misuse?
☐ Yes ☐ No ☐ Prefer not to answer

4. What is your sex?
☐ Male ☐ Female ☐ Non-binary ☐ Prefer not to answer

5. With which race or ethnicity do you most closely identify?
☐ White ☐ Black or African American ☐ American Indian or Alaska Native ☐ Asian
☐ Hispanic or Latino(a) ☐ Native Hawaiian or Other Pacific Islander
☐ More than one race/ethnicity. Please specify: ______ ☐ Other. Please specify: ______ ☐ Prefer not to answer

6. Please enter your age in years: _________________

7. Please indicate the Alabama COUNTY (e.g., Lee County) in which you reside: __________________ [dropdown list]

II. CURRENT AND PAST UTILIZATION OF OPIOID SENSORS

8. Are you aware of any devices that can rapidly sense the presence of opioids in a substance in real-time (a rapid-alert opioid sensor device)?
☐ Yes ☐ No

9. ***[Display if Q8 "Yes" is selected]*** Which rapid-alert opioid sensor devices have you previously heard about? Please select all that apply.
☐ TruNarc ☐ Fentanyl tests strips (FTS) ☐ Unsure ☐ Other. Please specify: ____________

10. Have you ever utilized a rapid-alert opioid sensor device?
☐ Yes. Please specify which device(s): ___________ ☐ No

11. In the past 3 months, how frequently or infrequently did you utilize a rapid-alert opioid sensor device?
☐ Never ☐ Rarely ☐ Sometimes ☐ Often ☐ All the time

III. PREFERENCES, BARRIERS, AND FACILITATORS FOR A RAPID-ALERT OPIOID SENSOR

On a scale of 1 to 7, please indicate your level of agreement or disagreement with the following statements regarding the most important elements of a rapid-alert opioid sensor device in your workplace or home, where 1=strongly disagree and 7=strongly agree.

12. ***To me, the most important parts of a rapid-alert opioid sensor are:***

	**Strongly Disagree (1)**	**Disagree**	**Somewhat Disagree**	**Neutral**	**Somewhat Agree**	**Agree**	**Strongly Agree (7)**
Affordability	☐	☐	☐	☐	☐	☐	☐
Portability	☐	☐	☐	☐	☐	☐	☐
Sensitivity and specificity of the test	☐	☐	☐	☐	☐	☐	☐
Ability to detect opioids in a broad range of substances	☐	☐	☐	☐	☐	☐	☐
Ability to detect multiple types of opioids	☐	☐	☐	☐	☐	☐	☐
Ability to detect the amount of an opioid in a substance (potency and purity)	☐	☐	☐	☐	☐	☐	☐
Rapid detection time	☐	☐	☐	☐	☐	☐	☐
No contact with unidentified drug substances (a “no-contact” probe)	☐	☐	☐	☐	☐	☐	☐
Ability to remotely view results using a mobile app	☐	☐	☐	☐	☐	☐	☐
Ability to remotely view results using a computer-based web interface	☐	☐	☐	☐	☐	☐	☐
Coverage by patient health insurance	☐	☐	☐	☐	☐	☐	☐
Availability for purchase in pharmacies as an over-the-counter (OTC) product	☐	☐	☐	☐	☐	☐	☐
Availability for purchase in pharmacies with a physician’s prescription	☐	☐	☐	☐	☐	☐	☐
Availability in law enforcement offices	☐	☐	☐	☐	☐	☐	☐
Availability in hospital emergency departments	☐	☐	☐	☐	☐	☐	☐
Other. Please specify:	☐	☐	☐	☐	☐	☐	☐

13. For your job, in which circumstances would you use a rapid-alert opioid sensor device? Please select all that apply.
    - ☐ Ensuring patient adherence to opioid therapy
    - ☐ Monitoring patients remotely for signs of opioid misuse (“real-time” monitoring)
    - ☐ Checking unknown solid substances (e.g., powders, pills) for the presence of opioids
    - ☐ Checking biological fluids (e.g., urine or blood) for the presence of opioids
    - ☐ Point-of-care testing or in-field diagnosis of opioid intoxication
    - ☐ Other. Please specify: ____

☐ Not applicable to my job

14. What **form of device** would be most useful to you in your workplace? Please select all that apply.
    ☐ No-contact probe sensor ☐ Test strips ☐ Skin patches ☐ Other. Please specify: ________ ☐ N/A

15. What would be a **reasonable detection time** for a rapid-alert opioid sensor? Detection time is defined as the amount of time needed for the device to sense and display results regarding the presence, identity, and amount of opioids detected.
    ☐ 1 minute ☐ 2 minutes ☐ 5 minutes ☐ 15 minutes ☐ 30 minutes ☐ 45 minutes ☐ 60 minutes

16. What is the **MAXIMUM** you would pay to purchase a rapid-alert opioid sensor **device kit** to use in your workplace or home? The device kit may take the form of a probe sensor to identify unknown substances, test strips for point-of-care diagnosis, and/or skin patches for patient use. The device kit will include all technology needed to monitor and interpret results.
    ☐ < $100 ☐ $100 ☐ $200 ☐ $300 ☐ $400 ☐ $500 ☐ > $500

17. What is the **MAXIMUM** you would pay **per test** for a rapid-alert opioid sensor device? For example, if the device kit came with enough testing supplies (e.g., disposable test strips) for at least one month, what is the maximum you would pay per test?
    ☐ <$15/test ☐ $20/test ☐ $30/test ☐ $40/test ☐ >$40/test

18. How can a rapid-alert opioid sensor be best designed to meet the needs of your workplace, home, or community? What would make it easier to use?: ____________________

19. What design elements should a rapid-alert opioid sensor NOT include? What would make it more difficult to use in your workplace, home, or community?: ________________________

IV. FACTORS AFFECTING UTILIZATION OF A RAPID-ALERT OPIOID SENSOR

On a scale of 1 to 7, please indicate your level of agreement or disagreement with the following statements regarding factors that affect / would affect your use of a rapid-alert opioid sensor device in your workplace, where 1=strongly disagree and 7=strongly agree.

20. ***Usefulness of a rapid-alert opioid sensor device in your workplace:***

	**Strongly Disagree (1)**	**Disagree**	**Somewhat Disagree**	**Neutral**	**Somewhat Agree**	**Agree**	**Strongly Agree (7)**	**N/A**
I would find a rapid-alert opioid sensor useful in my job.	☐	☐	☐	☐	☐	☐	☐	☐
Using a rapid-alert opioid sensor would enable me to accomplish tasks more quickly.	☐	☐	☐	☐	☐	☐	☐	☐
Using a rapid-alert opioid sensor would increase my productivity.	☐	☐	☐	☐	☐	☐	☐	☐

21. ***Ease of using a rapid-alert opioid sensor in your workplace:***

	**Strongly Disagree (1)**	**Disagree**	**Somewhat Disagree**	**Neutral**	**Somewhat Agree**	**Agree**	**Strongly Agree (7)**	**N/A**

My interaction with a rapid-alert opioid sensor would be clear and understandable.	☐	☐	☐	☐	☐	☐	☐	☐
It would be easy for me to become skillful at using a rapid-alert opioid sensor.	☐	☐	☐	☐	☐	☐	☐	☐
I would find a rapid-alert opioid sensor easy to use.	☐	☐	☐	☐	☐	☐	☐	☐
Learning to operate a rapid-alert opioid sensor would be easy for me.	☐	☐	☐	☐	☐	☐	☐	☐

22. ***Social factors influencing rapid-alert opioid sensor utilization in your workplace:***

	**Strongly Disagree (1)**	**Disagree**	**Somewhat Disagree**	**Neutral**	**Somewhat Agree**	**Agree**	**Strongly Agree (7)**	**N/A**
People who influence my behavior would think that I should use a rapid-alert opioid sensor.	☐	☐	☐	☐	☐	☐	☐	☐
People who are important to me would think that I should use a rapid-alert opioid sensor.	☐	☐	☐	☐	☐	☐	☐	☐
My professional colleagues would be helpful in the use of a rapid-alert opioid sensor.	☐	☐	☐	☐	☐	☐	☐	☐
In general, my workplace would support the use of a rapid-alert opioid sensor.	☐	☐	☐	☐	☐	☐	☐	☐

23. ***Resources affecting rapid-alert opioid sensor utilization in your workplace:***

	**Strongly Disagree (1)**	**Disagree**	**Somewhat Disagree**	**Neutral**	**Somewhat Agree**	**Agree**	**Strongly Agree (7)**	**N/A**
I would have the resources necessary to use a rapid-alert opioid sensor.	☐	☐	☐	☐	☐	☐	☐	☐
I would have the knowledge necessary to use a rapid-alert opioid sensor.	☐	☐	☐	☐	☐	☐	☐	☐

A rapid-alert opioid sensor would not be compatible with other systems I use. [r]	☐	☐	☐	☐	☐	☐	☐	☐
A specific person (or group) would be available for assistance with rapid-alert opioid sensor difficulties.	☐	☐	☐	☐	☐	☐	☐	☐

[r] Reverse-coded item.

24. ***Concerns regarding rapid-alert opioid sensor utilization in your workplace:***

	**Strongly Disagree (1)**	**Disagree**	**Somewhat Disagree**	**Neutral**	**Somewhat Agree**	**Agree**	**Strongly Agree (7)**	**N/A**
I would feel apprehensive about using a rapid-alert opioid sensor.	☐	☐	☐	☐	☐	☐	☐	☐
I would hesitate to use a rapid-alert opioid sensor for fear of making mistakes I cannot correct.	☐	☐	☐	☐	☐	☐	☐	☐
A rapid-alert opioid sensor would be somewhat intimidating to me.	☐	☐	☐	☐	☐	☐	☐	☐

25. ***Intentions to utilize a rapid-alert opioid sensor in your workplace:***

If a new rapid-alert opioid sensor was released on the market today…	**Strongly Disagree (1)**	**Disagree**	**Somewhat Disagree**	**Neutral**	**Somewhat Agree**	**Agree**	**Strongly Agree (7)**	**N/A**
I intend to use a rapid-alert opioid sensor in the next 3 months.	☐	☐	☐	☐	☐	☐	☐	☐
I predict I will use a rapid-alert opioid sensor in the next 3 months.	☐	☐	☐	☐	☐	☐	☐	☐
I plan to use a rapid-alert opioid sensor in the next 3 months.	☐	☐	☐	☐	☐	☐	☐	☐

Thank you so much for your time spent completing this survey! Please click “NEXT” to submit your responses and be re-directed to a separate questionnaire to collect your contact information so that we may enter you into a raffle to win one of three $100 Amazon electronic gift cards. The questionnaire will also ask about your willingness to be contacted for a follow-up interview. Your contact information will be used for gift card purposes and to schedule a follow-up interview (if indicated) only, and will not be connected to your survey responses. Your survey responses will remain anonymous.

Click “NEXT” to submit your survey responses.
